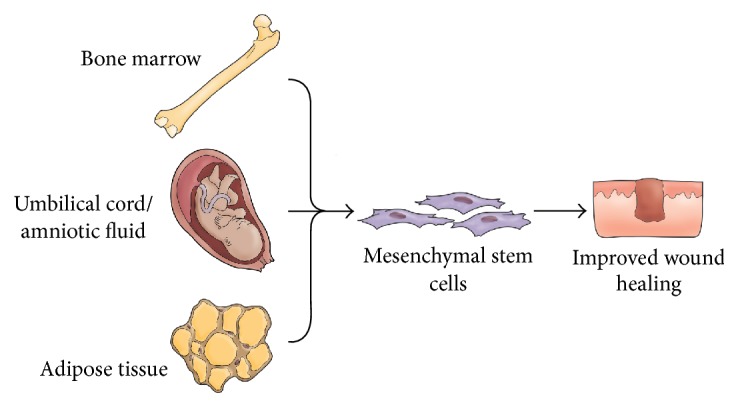# Mesenchymal Stromal Cells as Cell-Based Therapeutics for Wound Healing

**DOI:** 10.1155/2016/4157934

**Published:** 2016-02-04

**Authors:** Samir Malhotra, Michael S. Hu, Clement D. Marshall, Tripp Leavitt, Alexander T. M. Cheung, Jennifer G. Gonzalez, Harleen Kaur, H. Peter Lorenz, Michael T. Longaker

**Affiliations:** ^1^Hagey Laboratory for Pediatric Regenerative Medicine, Department of Surgery, Division of Plastic and Reconstructive Surgery, Stanford University School of Medicine, Stanford, CA 94305, USA; ^2^Institute for Stem Cell Biology and Regenerative Medicine, Stanford University School of Medicine, Stanford, CA 94305, USA; ^3^Department of Surgery, John A. Burns School of Medicine, University of Hawaii, Honolulu, HI 96813, USA

## Abstract

Chronic wounds are a source of substantial morbidity for patients and are a major financial burden for the healthcare system. There are no current therapies that reliably improve nonhealing wounds or reverse pathological scarring. Mesenchymal stromal cells (MSCs) are a promising source of novel cell-based therapies due to the ease of their harvest and their integral role in the native wound repair process. Recent work has addressed the problems of loss of plasticity and off-target delivery through use of modern bioengineering techniques. Here we describe the applications of MSCs harvested from different sources to the wound healing process and recent advances in delivery of MSCs to targeted sites of injury.

## 1. Introduction

Current management of chronic wounds is predominantly supportive and is often unable to prevent poor patient outcomes. Every year greater than $25 billion is spent on the treatment of chronic wounds in the United States [1]. Chronic wounds typically manifest in the elderly or those afflicted by diabetes mellitus or peripheral arterial disease. These wounds are characterized by a disruption in the critical process of neovascularization and resulting inhibition of normal tissue regeneration and repair [2]. Traditional methods to treat chronic wounds, such as physical debridement and administration of antibiotics, are often ineffective at aiding wound healing and act primarily to prevent infection of the wound [3]. On the other end of the wound healing spectrum is pathologic overhealing in the form of hypertrophic scars and keloids. These scars often cause pruritis and pain, and large scars, especially those over joints and on the face, can be a source of physical disability, psychological stress, and social stigma [4]. Currently, there are no reliable cell-based therapies to completely prevent deep dermal wounds from causing hypertrophic scars or keloids [5]. Consequently, there is an urgent need for alternative therapies to address the rising incidence of chronic wound-related health issues. Previous work has demonstrated that mesenchymal stromal cells (MSCs) play a central role in the wound healing process [6]. It is therefore necessary to assess the viability of cell-based therapeutics using MSCs derived from bone marrow, umbilical cord and amniotic fluid, and adipose tissue for nonhealing wounds and scars (Figure 1). Here we review key mechanisms and therapeutic properties of MSCs isolated from different sources for wound healing.

## 2. Identification and Isolation of Mesenchymal Stromal Cells

MSCs are typically defined as multipotent stromal cells that can differentiate into all cells of mesodermal origin, such as adipocytes, osteoblasts, chondrocytes, skeletal myocytes, and visceral stromal cells. MSCs are commonly sourced from bone marrow [7], but alternatively successful isolation of MSCs has been demonstrated from adipose tissue [8], umbilical cord (Wharton's jelly) [9], amniotic fluid [10], and synovial membrane [11]. MSCs from all of these sources share a similar set of core markers and properties, making them easily identifiable and accessible for use as therapeutics in clinical settings. Established criteria define MSCs as positive for CD105, CD73, and CD90 and negative for CD45, CD34, CD14 or CD11b, CD79*α* or CD19, and HLA-DR surface markers, and their ability to adhere to plastic [12]. It is important to note that MSCs are not a homogenous population and consist of many unique subpopulations that possess different cell surface markers and unique cellular properties. Moreover, while several combinations of markers can be used to identify populations of MSCs, selection efficiency can vary dependent on marker selection and tissue source [13]. Thus, in addition to these aforementioned general criteria, a diverse array of biomarkers has been identified to further characterize potential MSCs specific to their tissue of origin. For example, MSCs derived from bone marrow (BM-MSCs) have been demonstrated to express neural ganglioside GD2, a single surface marker that allows researchers to distinguish BM-MSCs from surrounding marrow elements [14]. Similarly, MSCs derived from white adipose tissue (Ad-MSCs) can express different characteristics if they were sourced from visceral fat or subcutaneous fat. Recent work demonstrates that presence of CD10 cell surface marker further identifies MSCs as subcutaneous fat-derived Ad-MSCs and presence of CD200 cell surface marker identifies cells as visceral fat-derived Ad-MSCs [15]. This existing knowledge of highly specific biomarkers for different types of MSCs allows them to be derived from a variety of tissue sources with a high degree of specificity, underscoring the potential of MSCs as an effective cell-based therapeutic.

## 3. Sources of Mesenchymal Stromal Cells

BM-MSCs are widespread in the marrow of trabecular bones and have been shown to be easily expandable and highly multipotent in nature. However, previous work with human BM-MSCs (BM-hMSCs) has demonstrated that BM-hMSCs harvested from older donors exhibit a decreased maximal life span and decreased proliferative capacity compared to BM-hMSCs harvested from younger donors [16]. This characteristic highlights a significant weakness for the use of BM-hMSCs as an autologous therapy for older patients.

Compared to BM-hMSCs, human Ad-MSCs (Ad-hMSCs) have similar morphology and possess the same potential to differentiate into any cell of mesodermal origin. It has been demonstrated that BM-hMSCs possess a greater capability for chondrogenic and osteogenic differentiation when compared to Ad-hMSCs, but BM-hMSCs have a greater probability for experiencing growth arrest [17]. Additionally, Ad-hMSCs can be more easily accessed using a simple surgical procedure and isolated through an uncomplicated, enzyme-based procedure. Furthermore, adipose tissue is known to contain the greatest frequency of MSCs, making it a highly viable source for autologous cell-based therapeutics and an alternative to bone marrow [18].

Additionally, MSCs can also be derived from Wharton's jelly cells of the human umbilical cord (WJ-hMSCs). Previous work has shown that these WJ-hMSCs have greatly reduced ability for adipogenic differentiation when compared to adult MSCs. However, the more primitive nature of WJ-hMSCs underlies their significantly greater proliferation capability and increased* ex vivo* expansion ability when compared to Ad-hMSCs and BM-hMSCs [19]. Thus, WJ-hMSCs represent an exciting alternative to traditional sources of MSCs, and our ability to harmlessly harvest WJ-hMSCs from the umbilical cord provides the potential for banking cells for future autologous, therapeutic use. Similarly, MSCs isolated from human amniotic fluid (AF-hMSCs) in both the second and third trimesters of pregnancy present another alternative source of MSCs for potential cell-based therapeutics [20]. AF-hMSCs exhibit the same phenotype and multipotent differentiation capability as BM-hMSCs, but they have significantly greater expansion potential when compared to BM-hMSCs [21]. Thus, it is clear that the variety of sources and differing properties of MSCs provide enormous scope for development of cell-based clinical therapeutics for wound healing.

## 4. Role of Mesenchymal Stromal Cells in the Wound Environment

Angiogenesis is a key physiological process that occurs during the wound healing cascade. During wound repair there is an explosive surge in neovascularization and angiogenesis, which creates a microvascular network within the granulation tissue in the wound site. MSCs are known to play an integral role in driving angiogenesis by secreting signaling/growth factors and also directing cell-to-cell interaction. Both* in vivo* and* in vitro* experiments have identified the ability of MSCs to release vascular endothelial growth factor (VEGF) and hypoxia inducible factor 1a (HIF1a) [22]. These key factors play a critical role in driving angiogenesis levels during wound healing [23]. Failure to maintain angiogenesis during wound repair is known to lead to chronic wound development [24].

Moreover, MSC activity has been reported throughout the inflammation, proliferation, and remodeling phases of wound repair. In the inflammatory phase, MSCs play a significant role in allowing the wound to advance past the inflammatory phase and into the proliferation phase of wound repair. Failure to correctly exit the inflammatory phase results in excessive scar production. Previous work has demonstrated that MSCs directly attenuate the inflammatory response in the wound environment by increasing the production of anti-inflammatory factors, such as interleukin-10 (IL-10), IL-4, and transforming growth factor-*β* (TGF-*β*). MSCs also decrease local production of common proinflammatory factors such as tumor necrosis factor-*α* (TNF-*α*) and interferon-*γ* [25]. Additionally, MSCs can effectively suppress the recruitment and proliferation of activated T-cells responding to the injury. Therefore, it is likely that the anti-inflammatory properties of MSCs play a critical role in allowing the wound to commit to the next stage of wound repair and avoid becoming a chronic, nonhealing wound [26].

Signals generated by chemotactic proteins cause MSCs to migrate to sites of injury, a response that is mediated by the activation of matrix metalloproteinases [27–29]. Once MSCs have arrived at the site of injury they may improve wound healing in several ways. There is experimental evidence that MSCs possess antimicrobial properties and may help to prevent infections in the wound bed. MSCs can secrete bactericidal factors, such as LL-37, directly into the local environment, and they can indirectly increase the amount of phagocytosis by activating immunomodulatory factors [30]. MSCs can improve tissue repair through increased secretion of growth factors, such as epidermal growth factor (EGF), keratinocyte growth factor, insulin-like growth factor-1 (IGF-1), and vascular endothelial growth factor-*α* (VEGF-*α*) [31]. Finally, MSCs have demonstrated the ability to influence other cells in the local environment to further enhance tissue repair. For example, the presence of MSCs increases production of type I collagen, which is thought to increase tensile strength of the wound and alter gene expression in dermal fibroblasts [32].

## 5. Therapeutic Uses for MSCs

Early animal experiments with BM-hMSCs demonstrate the capability to dramatically increase the rate of wound healing in a splinted murine full thickness wound model. Xenografted BM-hMSCs promote significantly higher rates of wound closure and the capability to enhance repair of both local and distant wounds: healing is improved in both graft sites and nongrafted wounds. This suggests a systemic response that is characterized by the recruitment of existing host MSCs to the site of injury. Furthermore, xenografted BM-hMSCs result in significant elevation of Wnt3a, VEGF, and platelet-derived growth factor-*α* (PDGF-*α*) in the wound bed, which then alter endogenous fibroblast and endothelial cell activity. Consequently, repair initiated by engrafted BM-hMSCs activates endogenous signaling pathways that allow repair to continue even without the presence of engrafted cells. The increased presence of proangiogenic factors after BM-hMSC transplantation suggests that BM-hMSCs could be utilized to rescue deficient angiogenesis observed in chronic wound environments [33]. However, it is important to note that wounds supplemented with whole bone marrow have demonstrated increased healing capabilities compared to wounds transplanted with only BM-MSCs; this suggests that supportive factors from the niche of BM-MSCs could further supplement the healing capability of BM-MSCs [34]. Although the* in vivo* therapeutic potential of BM-MSCs is well established in mouse models, low viability of transplanted cells inhibits widespread clinical adoption.

Consequently, focus has shifted towards developing bioengineering strategies to increase the effectiveness of BM-hMSC delivery. For example, gelatin microspheres were seeded with BM-hMSCs and EGF to create an engineered skin construct. This method of delivery resulted in significantly accelerated wound healing but, more importantly, seemed to form a favorable microenvironment for cell differentiation and stratification [35]. Additionally, very recent work has demonstrated that BM-hMSC-derived exosomes, which are small, membrane-bound vesicles, are able to enhance the migration of chronic wound fibroblasts and induce angiogenesis* in vitro*. These exosomes are protected from proteolytic degradation in the wound environment by a lipid bilayer shell, allowing them to effectively transfer signals and regulatory components, such as miRNAs and mRNAs, to target cells. Although experimentation is in its early stages, BM-hMSC-derived exosomes hold immense potential for a novel therapy for chronic wounds [36]. Moreover, another recent murine study has demonstrated the potential of BM-hMSCs delivered with fibrin glue to significantly increase the quality of healed skin in chronic wound conditions [37]. Thus, BM-hMSCs show great promise in a variety of preclinical injury models, but there is still a need for more rigorous research on their abilities in clinical settings [38]. Clinical studies with BM-hMSCs have resulted in immense increases in wound healing capability. Early efforts demonstrate that BM-hMSC application with a fibrin spray results in chronic wound healing that is correlated with the number of cells applied [39]. A later trial demonstrated that injection of BM-hMSCs into chronic wounds treated with a collagen sponge composite graft resulted in a 90% healing rate of chronic wounds in a study of 20 patients [40]. Thus, both preclinical and clinical data have shown strong support for the continued development of BM-hMSC-based therapies for chronic wounds.

MSCs isolated from the umbilical cord and amniotic fluid are another promising source of wound healing therapies. Recent work demonstrates the ability to effectively isolate human MSCs from Wharton's jelly of the umbilical cord (WJ-hMSCs). These MSCs increase the expression of key wound healing genes via paracrine signaling and also significantly enhance the rate of wound repair in a murine model. Notably, the harvest of WJ-hMSCs is neither painful nor invasive and has proven to be a more efficient source of stromal cells when compared to BM-MSCs due to a higher capacity for proliferation. This method could also feasibly allow patients to bank stromal cells for future treatments with autologous cells [41]. Additionally, recent work with animal models has shown that application of WJ-hMSCs together with poly(vinyl alcohol) hydrogel (PVA) membrane significantly accelerates chronic wound healing by driving skin regeneration and decreasing ulcerated areas [42].

Moreover, AF-MSCs have shown greater ability to upregulate multiple angiogenic factors, such as IGF-1, EGF and IL-8, when compared with MSCs derived from other sources. Preclinical animal model studies have shown the ability of human AF-hMSCs to promote reepithelialization and increase overall rate of wound repair. Moreover, AF-hMSCs demonstrate high rates of engraftment and do not express class II major histocompatibility complex, making them a highly viable candidate for both autologous and allogeneic cell-based therapies [43].

Finally, Ad-MSCs have demonstrated the ability to significantly increase the overall rate of wound repair in preclinical murine models [44, 45]. Injection of Ad-MSCs in diabetic murine wound healing models demonstrates the ability of Ad-MSCs to significantly increase the expression of VEGF and levels of angiogenesis in the wound bed. These preclinical, animal model results suggest that Ad-MSCs have tremendous potential to rescue deficient angiogenesis mechanisms in chronic wounds [46]. Recent work has shown that allogeneic transplantation of Ad-MSCs alongside artificial skin into full thickness wounds of diabetic mice results in significantly increased levels of vascularization and wound healing [47]. These findings provide tremendous scope for artificial skin transplantation for diabetic patients whose autologous skin transplants fail to engraft in chronic wounds. Furthermore, modifying Ad-MSCs with surface carriers that have medical-grade silicone coated by plasma polymerization with a thin layer of acrylic has been shown to successfully increase the efficiency of Ad-MSC delivery to wounds and ultimately increase the rate of wound healing [48].

Further studies with human Ad-hMSCs have demonstrated increased angiogenesis and wound healing in murine models via paracrine mechanisms [49]. A clinical trial examined the transplantation of autologous Ad-MSCs differentiated into adipocytes as a therapy for depressed scars; 12 weeks after operation, clinicians were able to establish the safety and efficacy of transplantation for treatment of depressed scars [50]. Thus the combination of preclinical and clinical evidence provides strong support for further exploration of Ad-hMSC-based therapeutics for conditions such as chronic wounds. However,* in vivo* data has shown that Ad-hMSCs promote the proliferation of breast, prostate, and sarcomatous cancer cells after transplantation [51]. Consequently, while the use of Ad-hMSCs as a clinical therapeutic seems tremendous, further studies are still needed to determine the safety of Ad-hMSC transplants in humans.

## 6. Conclusions

Chronic nonhealing wounds are a tremendous burden for patients and the healthcare system. Current wound treatment is ineffective in many cases, so it is imperative that alternative sources of therapy are explored. MSCs are an easily accessible source of cells for potential cell-based therapies, and they have demonstrated tremendous ability in preclinical animal models to improve wound repair outcomes. Thus, MSC-based therapies warrant further development and exploration with large-scale clinical trials.

## Figures and Tables

**Figure 1:**